# Immune checkpoint inhibitor therapy is associated with a decreased risk of developing melanoma brain metastases

**DOI:** 10.1038/s44276-025-00137-2

**Published:** 2025-04-11

**Authors:** Anna Fager, Matilda Samuelsson, Roger Olofsson Bagge, Aldina Pivodic, Sara Bjursten, Max Levin, Henrik Jespersen, Lars Ny

**Affiliations:** 1 Department of Oncology, Sahlgrenska University Hospital, Gothenburg, Sweden; 2 Department of Oncology, Institute of Clinical Sciences, Sahlgrenska Academy, University of Gothenburg, Gothenburg, Sweden; 3 Department of Surgery, Institute of Clinical Sciences, Sahlgrenska Academy, University of Gothenburg, Gothenburg, Sweden; 4 Department of Surgery, Sahlgrenska University Hospital, Gothenburg, Sweden; 5 APNC Sweden, Mölndal, Sweden; 6 Department of Oncology, Oslo University Hospital, Oslo, Norway

## Abstract

**Background:**

Despite recent advancements in metastatic melanoma treatment, the emergence of melanoma brain metastases (MBM) continues to pose a challenge. This study aimed to explore factors associated with MBM development.

**Methods:**

This retrospective study included patients diagnosed with advanced melanoma (unresectable stages III and IV [M1a-c]) between 2013 and 2019 at Sahlgrenska University Hospital, Gothenburg, Sweden. Differences in baseline and primary tumor characteristics, mutational status, biomarker levels, and first-line treatment between patients who developed MBM (BM+) and patients who did not develop MBM (BM-) were analyzed using univariable and multivariable Cox proportional hazard regression.

**Result:**

Of 395 patients, 91 subsequently developed MBM. Patients who received immune checkpoint inhibitors (ICI) as first-line treatment had a reduced risk of MBM development (*p* ≤ 0.001). None of the eleven patients who received CTLA-4 inhibitors as monotherapy or in combination with PD-1 inhibitors as first-line treatment developed brain metastases. Elevated plasma levels of S100B (*p* = 0.021) and higher metastatic stage (*p* = 0.047) were also associated with an increased risk of MBM development.

**Conclusion:**

ICI treatment is associated with a decreased risk of MBM development, suggesting a protective role. Elevated S100B levels and stage IV disease at advanced melanoma diagnosis might indicate an increased risk of MBM development.

## Introduction

Melanoma is among the cancers with the highest risk of developing brain metastases (BM), with a reported incidence of over 40% in metastatic setting [1, 2]. Historically, melanoma brain metastases (MBM) have been associated with limited treatment options and a poor prognosis [1, 3–5]. The approval of immune checkpoint inhibitors (ICI), such as cytotoxic T-lymphocyte-associated antigen 4 (CTLA-4) inhibitors and programmed cell death protein 1 (PD-1) inhibitors, and targeted therapies like BRAF and MEK inhibitors in the last decades, have improved overall survival (OS), with long-lasting antitumoral effects documented in patients with stage IV melanoma [6–13]. Phase 2 trials have further demonstrated promising outcomes in patients with MBM, including intracranial response rates reaching 55% and 1-year survival exceeding 80% with combined CTLA-4 (ipilimumab) and PD-1 (nivolumab) inhibitors [14]. Similar response rates have been reported with combined BRAF (dabrafenib) and MEK (trametinib) inhibitors, although the responses are rarely durable [15]. Despite these advances, not all patients with MBM are suitable candidates for these novel systemic therapies, and many still face a poor prognosis. Early diagnosis of metastatic disease and prompt initiation of antitumoral treatment are vital to improve outcomes.

Several factors have been reported as associated with an increased risk of MBM development. These include primary tumor location on the scalp [16, 17] or head/neck [18–21], nodular primary tumor subtype [16, 20], primary tumor ulceration [20–24], Breslow thickness exceeding 4 mm [16, 19], positive BRAF V600 mutation status [25–27], higher metastatic stage [20, 28–30], and elevated plasma levels of lactate dehydrogenase (LDH) [28]. Interestingly, first-line systemic treatment in patients with advanced melanoma could potentially also influence the risk of later MBM development. The use of ICI in the first line has been associated with a decreased risk of MBM development, and a later onset of MBM has been reported also in patients with BRAF mutations who received ICI in the first line compared with targeted therapy [31–33]. Still, other studies have not identified significant differences between different treatment modalities [26, 34].

The aim of this study was to further investigate factors predicting MBM development in patients with advanced melanoma in the era of novel systemic therapies.

## Methods

This is a retrospective single-center study performed at the Department of Oncology, Sahlgrenska University Hospital in Gothenburg, Sweden, from January 1st 2013 to June 30th 2019.

### Patients

All patients with advanced melanoma, defined as unresectable stages III or IV (M1a-c) cutaneous melanoma disease, were identified from the oncology outpatient clinic using the International Classification of Diseases (ICD)-10 codes C43.1–9 or Z85.8 C. Subsequently, the cohort was divided into patients who developed BM (BM+) and those who did not (BM-) during the follow-up period; the former group of patients was identified using the ICD-10 code C79.3. The data cut-off point was set to December 31st 2019, ensuring a minimum follow-up of six months. Inclusion criteria were advanced melanoma diagnosis within the specified time frame. Patients with MBM at time of diagnosis of advanced melanoma were excluded. Brain imaging was performed at baseline using either computer tomography (CT) or magnetic resonance imaging (MRI). If negative CT scan was retrieved a MRI scan was performed if clinically indicated. After initiation of therapy, patients underwent routine brain imaging at yearly intervals. For patients who developed symptoms suggestive of BM, MRI scans were used to detect potential metastasis development. Eligible patients had accessible medical records at the Department of Oncology, Sahlgrenska University Hospital, Gothenburg, Sweden. Baseline and primary tumor characteristics, mutation status, levels of melanoma biomarkers, and data regarding metastatic patterns and first-line treatment at the time of advanced melanoma diagnosis were collected for both groups. Choice of first-line treatment was determined on a recommendation at a multidisciplinary conference with a final decision made by the attending physician.

### Objectives

The primary objective was to evaluate the impact of first-line treatment administered at advanced melanoma diagnosis on MBM development. Secondary objectives were to identify other factors influencing MBM development, including baseline and primary tumor characteristics, level of melanoma biomarkers, and stage at advanced melanoma diagnosis.

### Assessments

Staging was done according to the 8th edition of the American Joint Committee on Cancer staging manual [35]. Baseline clinical characteristics were reported at the time of advanced melanoma diagnosis. Plasma levels of biomarkers LDH and S100B were considered elevated if they exceeded the upper limit of normal as per local cut-off values.

### Statistical analyses

Continuous variables were described by medians and interquartile ranges (IQR) and categorical variables by counts and percentages. To evaluate predictive factors for MBM development, univariable Cox proportional hazard regression analyses were performed. The assumption of proportional hazards was tested and found satisfactory for all predictors. Hazard ratios (HR) with 95% confidence intervals (CI) are reported with *p* values, whereby *p* < 0.05 are considered statistically significant. Variables with significant associations (*p* < 0.05) in the univariable analyses were studied as potential variables in the multivariable analyses. The multivariable model was selected by using stepwise forward regression to obtain independently significant predictors. The cumulative incidence of MBM development overall and by variables selected for the multivariable analyses were calculated by adjusting for death as a competing risk.

The follow-up duration was calculated from the date of advanced melanoma diagnosis to the date of MBM diagnosis, death, or censored at study termination, and was described by median and IQR. OS was calculated from the date of advanced melanoma diagnosis to the date of death or censored and estimated by the Kaplan–Meier method. To evaluate the difference in OS between BM+ and BM- patients, a time-updated Cox regression analysis was performed, handling a MBM event as a time-updated variable, to take immortal time bias into consideration. For descriptive purposes, OS was calculated for BM+ and BM- separately, without taking immortal time bias into account. Both groups were followed up from baseline. In cases where the exact date of advanced melanoma diagnosis was missing, the date was set at the start of first-line treatment. The median duration of CTLA-4 inhibitor +/− PD-1 inhibitor therapy was calculated from date of treatment start to date of last treatment given. The median time to next treatment was calculated from date of CTLA-4 inhibitor +/- PD-1 inhibitor therapy termination to date of next treatment start or censored, whichever came first. Statistical analyses were performed in SPSS version 29.0.0.1 and R Studio, using version 4.3.2 of R.

## Results

**Fig. 1:**
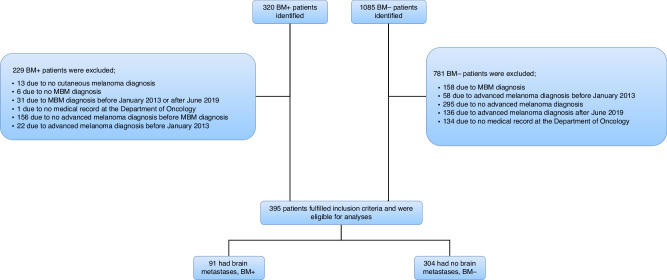
Flowchart illustrating the patient inclusion process. BM Brain Metastases, MBM Melanoma Brain Metastases.

**Fig. 2:**
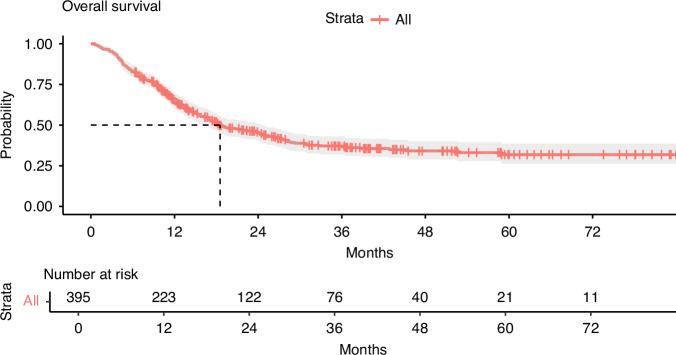
OS of all patients. Median OS was 18.5 months (IQR 9.0-31.7). OS at 12-month follow-up was 64% (95% CI 0.60 to 0.69). OS overall survival.

Of 320 BM+ and 1085 BM- patients identified, 91 (28.4%) and 304 (28.0%) patients met the inclusion criteria, respectively, and were included in the analyses corresponding to development of BM in 23.0% of patients (Flowchart, Fig. 1). The median follow-up duration was 12.4 months (IQR 7.5–28.8). The median OS was 18.5 months (IQR 9.0–31.7) and 64% (95% CI 0.60–0.69) of the patients were alive at the 12-month follow-up (Fig. 2). Patients that developed MBM had a shorter OS compared to those who did not (HR 7.19, 95% CI 5.41–9.56, *p* < 0.001), median OS 13.9 months (IQR 9.3–21.7) versus 27.9 months (IQR 8.9–35.4).

Baseline clinical and primary tumor characteristics are presented in Table 1. The median age was 69 years and was slightly higher in patients without MBM (70 vs. 64 years). Of the included patients, 57% were men, 41% had a BRAF mutation, 37% had elevated levels of LDH, and 48% had elevated levels of S100B. The cumulative incidence of MBM overall was 17.4% (95% CI 13.8–21.4%) at the 12-month follow-up.

**Table 1:** Baseline and primary tumor characteristics.

Variable	Characteristic	All N (%)	BM + N (%)	BM- N (%)
Patients		395 (100)	91 (100)	304 (100)
Sex	Female	171 (43.3)	36 (39.6)	135 (44.4)
	Male	224 (56.7)	55 (60.4)	169 (55.6)
T-stage	T1	34 (8.6)	5 (5.5)	29 (9.5)
	T2	69 (17.5)	27 (29.7)	42 (13.8)
	T3	74 (18.7)	13 (14.3)	61 (20.1)
	T4	128 (32.4)	23 (25.3)	105 (34.5)
	Missing^a^	90 (22.8)	23 (25.3)	67 (22.0)
Ulceration	No	103 (26.1)	17 (18.7)	86 (28.3)
	Yes	150 (38.0)	36 (39.6)	114 (37.5)
	Missing^a^	142 (35.9)	38 (41.8)	104 (34.2)
Site	Head/neck	56 (14.2)	7 (7.7)	49 (16.1)
	Torso	142 (35.9)	34 (37.8)	108 (35.5)
	Upper limb	37 (9.4)	4 (4.4)	33 (10.9)
	Lower limb	89 (22.5)	24 (26.5)	65 (21.4)
	Missing^a^	71 (18.0)	24 (24.2)	49 (16.1)
Mutation status	Other	202 (50.1)	39 (42.9)	163 (53.6)
	BRAF	162 (41.0)	49 (53.8)	113 (37.2)
	Missing	31 (7.8)	3 (3.3)	28 (9.2)
Age^b^—Median (IQR)		69 (58–77)	64 (55–74)	70 (60–78)
Age^b^	≤65	167 (42.3)	51 (56.0)	116 (38.2)
	>65	228 (57.7)	40 (44.0)	188 (61.8)
Stage^b^	Unresectable III	49 (12.4)	4 (4.4)	45 (14.8)
	IV (M1a)	56 (14.2)	10 (11.0)	46 (15.1)
	IV (M1b)	103 (26.1)	22 (24.2)	81 (26.6)
	IV (M1c)	187 (47.3)	55 (60.4)	132 (43.4)
PS (ECOG)^b^	0–1	229 (58.0)	44 (48.4)	185 (60.9)
	≥2	52 (13.2)	11 (12.1)	41 (13.5)
	Missing	114 (28.9)	36 (39.6)	78 (25.7)
Level of LDH^b^	Normal	170 (43.0)	30 (33.0)	140 (46.1)
	Elevated	144 (36.5)	34 (37.4)	110 (36.2)
	Missing	81 (20.5)	27 (29.7)	54 (17.8)
Level of S100^b^	Normal	120 (30.4)	18 (19.8)	102 (33.6)
	Elevated	188 (47.6)	45 (49.5)	143 (47.0)
	Missing	87 (22.0)	28 (30.8)	59 (19.4)
First-line tx^b^	ICI	152 (38.5)	17 (18.7)	135 (44.4)
	PD-1 inhibitors	141 (35.7)	17 (18.7)	124 (40.8)
	CTLA-4 inhibitors	8 (2.0)	0 (0.0)	8 (2.6)
	PD-1 + CTLA-4 inhibitors	3 (0.8)	0 (0.0)	3 (1.0)
	Chemotherapy	55 (13.9)	13 (14.3)	42 (13.8)
	Target therapy	86 (21.8)	27 (29.7)	59 (10.4)
	Local tx	70 (17.7)	23 (25.3)	47 (15.5)
	No tx	29 (7.3)	11 (11.0)	18 (5.9)
	Missing	3 (0.8)	0 (0.0)	3 (1.0)

*BM* Brain Metastases, *T-stage* Tumor stage, *IQR* Interquartile Range, *PS (ECOG)* Performance Status (European Cooperative Oncology Group), *LDH* Lactate Dehydrogenase, *tx* treatment, *ICI* Immune Checkpoint Inhibitors, *PD-1* programmed cell death 1, *CTLA-4* cytotoxic T-lymphocyte associated protein 4.

^a^missing data, primary tumor unknown, or multiple primary tumors.

^b^at advanced melanoma diagnosis.

Twenty-eight (30.8%) patients developed MBM during first-line treatment, 14 patients during targeted therapy, ten patients during PD-1 inhibitor therapy, and four patients during chemotherapy.

**Fig. 3:**
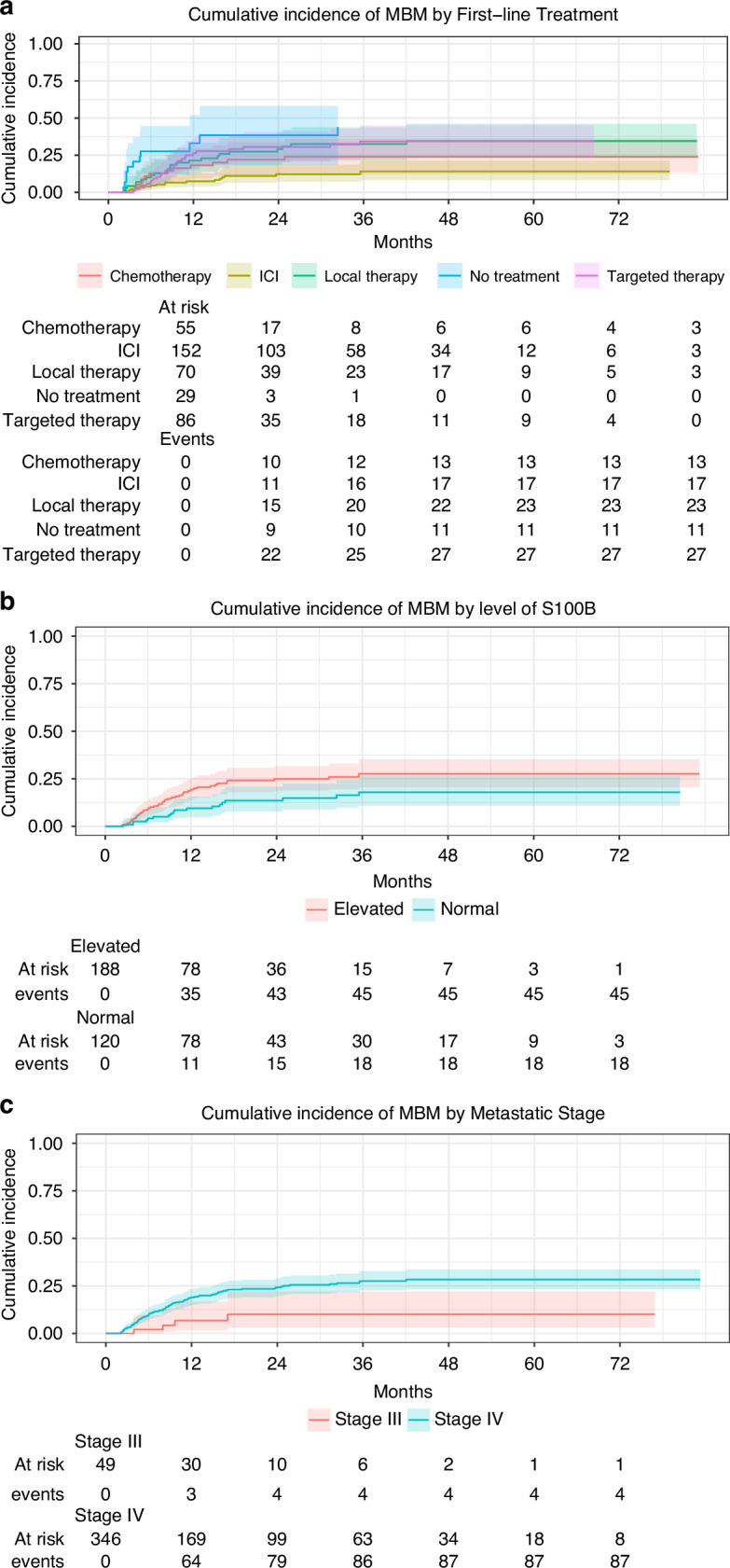
Cumulative incidence of MBM by selected subgroups. **a** Cumulative incidence of MBM by first-line treatment. **b** Cumulative incidence of MBM by level of S100B. **c** Cumulative incidence of MBM by metastatic stage. MBM melanoma brain metastases.

Results from the uni- and multivariable analyses are summarized in Table 2. In univariable analysis, factors associated with an increased risk of MBM development were *T*-stage at primary tumor diagnosis, metastatic stage, plasma levels of S100B, plasma levels of LDH, and first-line treatment at advanced melanoma diagnosis. The cumulative incidence of MBM development by first-line treatment is presented in Fig. 3a. Eleven patients received CTLA-4 inhibitors in the first line: eight had CTLA-4 inhibitor monotherapy and three had combined therapy with a PD-1 inhibitor. None of the eleven patients who received first-line CTLA-4 inhibitor monotherapy or combined therapy with a PD-1 inhibitor developed MBM. The median duration of CTLA-4 inhibitor +/− PD-1 inhibitor therapy was 63 days. In patients who received CTLA-4 inhibitor monotherapy, one patient had two infusions, and six patients had four infusions. In patients that had combined CTLA-4 inhibitor and PD-1 inhibitor therapy, one patient had one infusion, and one patient had four infusions of combined CTLA-4 inhibitor and PD-1 inhibitor therapy, followed by 55 infusions every second week of PD-1 inhibitors. The median time to next treatment in this subgroup of patients was 164 days. For two patients complete data regarding number of infusions and treatment durations are missing. Three of the 11 patients participated in the Checkmate 067 trial.

**Table 2:** Uni- and multivariable Cox regression analysis evaluating factors predicting MBM development.

				Univariable analysis			Multivariable analysis		
Variable	Characteristic	BM + *n*	BM- *n*	HR	95% CI	*p* value	HR	95% CI	*p* value
No of patients	N	91	304						
Sex	Female	36	135	Ref.					
	Male	55	169	1.36	0.90–2.08	0.15			
T-stage	T1	5	29	Ref.					
	T2	27	42	3.04	1.17–7.89	0.023			
	T3	13	61	1.46	0.52–4.09	0.47			
	T4	23	105	1.39	0.53–3.67	0.50			
Ulceration	No	17	86	Ref.					
	Yes	36	114	1.43	0.80–2.54	0.23			
Site	Head/neck	7	49	Ref.					
	Torso	34	108	2.07	0.92–4.67	0.080			
	Upper limb	4	33	0.83	0.24–2.85	0.77			
	Lower limb	24	65	2.06	0.89–4.77	0.093			
Mutation status	Other	39	163	Ref.					
	BRAF	49	113	1.43	0.94–2.18	0.097			
Age^a^	cont.	91	304	0.99	0.97–1.00	0.092			
Age^a^	≤65	51	116	Ref.					
	>65	4	188	0.68	0.45–1.03	0.065			
Stage^a^	Unresectable III	4	45	Ref.			Ref.		
	IV	87	259	3.32	1.22–9.06	0.019	2.78	1.02–7.63	0.047
PS (ECOG)^a^	0–1	44	185	Ref.					
	≥2	11	41	1.80	0.93–3.50	0.083			
Level of LDH^a^	Normal	30	140	Ref.					
	Elevated	34	110	1.95	1.19–3.19	0.008			
Level of S100^a^	Normal	18	102	Ref.			Ref.		
	Elevated	45	143	2.24	1.29–3.88	0.004	1.98	1.11–3.55	0.021
First line tx^a^	Immunotherapy	17	135	Ref.			Ref.		
	Target therapy	27	59	3.69	2.01–6.79	<0.001	2.86	1.53–5.37	0.001
	Chemotherapy	13	42	3.48	1.69–7.19	<0.001	3.37	1.63–7.00	0.001
	Local tx	23	47	3.49	1.86–6.54	<0.001	3.16	1.64–6.09	<0.001
	No tx	11	18	12.12	5.59–26.28	<0.001	11.24	5.14–24.59	<0.001

Missing data were included in the Cox proportional hazard regression analyses but not reported due to clinical irrelevance.

*BM* Brain Metastases, *T-stage* Tumor stage, *PS (ECOG)* Performance Status (European Cooperative Oncology Group), *LDH* Lactate Dehydrogenase, *tx* treatment, *ICI* Immune Checkpoint Inhibitors.

^a^at advanced melanoma diagnosis.

Along with first-line treatment, S100B plasma levels and metastatic stage at advanced melanoma diagnosis were independently selected in the multivariable model, which showed that patients treated with ICI in the first line had a reduced risk of MBM development compared with patients who received targeted therapy (HR 2.86, 95% CI 1.53–5.37, *p* = 0.001), chemotherapy (HR 3.37, 95% CI 1.63–7.00, *p* = 0.001), local therapy (HR 3.16, 95% CI 1.64–6.09, *p* < 0.001), and no treatment (HR 11.24, 95% CI 5.14–24.59, *p* < 0.001). Patients with elevated levels of S100B were more likely to develop MBM compared with patients with normal levels (HR 1.98, 95% CI 1.11–3.55, *p* = 0.021). Patients who presented with stage IV (M1a-c) melanoma had an increased risk of MBM development compared with patients who presented with unresectable stage III disease (HR 2.78, 95% CI 1.01–7.63, *p* = 0.047). The cumulative incidence of MBM development by S100B levels and by metastatic stage is presented in Fig. 3b, c, respectively.

## Discussion

This study suggests that first-line ICI treatment decreases the risk of later BM development in patients with advanced melanoma. Interestingly, none of the eleven patients who received first-line treatment with a CTLA-4 inhibitor, either as monotherapy or in combination with a PD-1 inhibitor, developed later MBM, which to our knowledge has not previously been reported [27, 36, 37]. The observation is interesting because CTLA-4 inhibition is considered important for response in BM. In addition, lymphocyte infiltration in the brain is a feature of patients with genetic CTLA-4 deficiency but has not been described in patients with genetic PD-1 deficiency. Collectively, these observations suggest that CTLA-4 inhibition promotes immune infiltration in the brain and could possibly prevent development of BM. Furthermore, patients receiving ICI treatment had a lower risk for MBM development compared with patients who received other first-line treatments, findings also supported by previous studies [31–33].

PD-1 inhibitor treatment has been reported to be superior to targeted therapy in terms of reduced frequency of MBM development [33]. A higher incidence of MBM was reported in patients with BRAF mutations following BRAF and MEK inhibitor treatment compared with combined PD-1 and CTLA-4 inhibitors [32]. In another report, PD-1 inhibitor treatment reduced the risk of MBM development by 70% [31].

In addition to first-line treatment, associations between other patient- and disease-related factors and the risk of MBM development were explored. The results of multivariable analyses showed that increased plasma levels of S100B at advanced melanoma diagnosis were independently associated with MBM development besides the first-line treatment. S100B is a protein primarily expressed by glial cells, especially astrocytes. S100B is released into the bloodstream upon traumatic brain injury and is used as a biomarker for brain damage [38]. While S100B is primarily a glial marker, it is also expressed by melanocytes, from which melanoma tumor cells originate, and its expression in melanoma reflects a cellular origin in the neural crest. Elevated levels of S100B have been identified in patients with metastatic melanoma and patients with tumor relapse from melanoma [39], and are associated with impaired OS [40–44]. Thus, S100B is a useful biomarker, which can be utilized as a prognostic tool and contribute to relapse detection in patients with melanoma. It can be hypothesized that S100B expression in melanoma is linked to dedifferentiation and accession of stem-like properties associated with increased neurotropism. Thus, the association between elevated S100B levels and an increased risk of MBM development might be related to a more aggressive tumor biology. To our knowledge, no other study evaluating the relationship between S100B and MBM development has been published, and further analyses are needed to confirm our results.

In our cohort, the clinical stage at advanced melanoma diagnosis (unresectable stage III compared to stage IV) was associated with MBM development. This finding is in line with those of previous reports. A study by Bedikian and co-workers (2011) identified an increased risk of MBM development in patients with M1b or M1c melanoma compared with stage III or M1a melanoma [28]. Although the study was performed before the introduction of ICI and targeted therapies and choice of subgroups were different from the ones in our study, the results are similar and indicate that stage at first advanced melanoma diagnosis might be a predictor of later MBM development. In addition, the finding of a cumulative incidence of MBM at 17.4% at 12 months follow-up suggests a need for radiologically MBM screening regularly in patients with advanced melanoma.

In a previous study, BRAF mutation status have been reported to be associated with MBM development. However, this could not be corroborated in the present study cohort, which could be related to the limitations of this study. Specifically, we did not include patients with MBM as the first metastatic site. While primary tumor location, subtype, and *T*-stage have previously been associated with MBM development [16–27], they were not identified as significant factors in the present study.

This study has several limitations. First, the data were retrospectively collected from medical records and the reporting of some variables was incomplete, leading to missing data. Documentation of the patient’s performance status at advanced melanoma diagnosis was not always consistent, levels of LDH and S100B were not available of all patients and some pathology reports were unavailable or incomplete. In light of the limitations of the retrospective design, a prospective study is suggested for further research. The prospective design would decrease the risk of missing data and might enable a more even distribution of first-line treatments and BRAF mutation status in the cohort.

The study strengths include the population-based cohort. All patients diagnosed with advanced melanoma from a total population of nearly two million individuals were considered for study inclusion, limiting selection bias. The real-world study design might provide insights not to be captured in clinical trials. The study period reflects the development of new systemic treatments for melanoma and illustrates the shift in treatment principles and patterns over the last decade. The potential prevention of MBM development as observed here is considered to be of major clinical benefit and warrants further investigations, providing additional data addressing BM development in different populations and cohorts.

## Conclusion

Our results suggest that ICI treatment is associated with a decreased risk of MBM development in patients with advanced melanoma and no BM at diagnosis. We also found that patients with elevated S100B plasma levels had a higher risk of MBM development, which should be considered at follow-up.

## Data Availability

Upon request discuss with authors.
